# Supplementary material for: Emerging Direct Targeting β-Catenin Agents
Source: Molecules. 2022 Nov 10;27(22):7735. doi: 10.3390/molecules27227735 (PMC9698307; doi:10.3390/molecules27227735)
Supplement: Supplementary file 1 [file molecules-27-07735-s001.zip › molecules-1986143-supplementary.pdf]

Table S1. Biological assays used to evaluate the β-catenin activity.

Entry	Compd	Assay
**1**	FH535	TOPFLASH luciferase activity
**2**	GW9662	TOPFLASH luciferase activity
**3–6**	FH535 analogs	Luciferase-based TOPFlash assay
**7**	MSAB	TOP-Luc activity assay
**8–9**	MSAB analog	TOPFlash assay
**10**	LF3	AlphaScreen
**11**	LF3 analog	AlphaScreen
**12–14**	iCRTs	Coimmunoprecipitation (coIP)
**15**	ZINC02092166	Wnt-responsive luciferase reporter assay
**16,17**	ZINC analogs	Wnt-responsive luciferase reporter assay
**18**	PNU-74654	ITC, NMR, Luciferase reporter system for Tcf-4 transactivation
**19**	UU-T01	AlphaScreen, fluorescence polarization (FP)
**20, 21**	UU-T02/03	Fluorescence polarization (FP)
**22**	HI-B1	β-catenin/TCF4 luciferase activity
**23–26**	PKF	Tcf4 fragment (residues 8–54) fused to glutathione-S-transferase (GST), anti-GST antibody, and alkaline phosphatase (AP)-conjugated secondary antibody
**27**	Henryn	Purified recombinant proteins β-catenin and TCF4 (0.5μg) were incubated with Henryin
**28**	BC21	FP-based assay developed for that purpose (unpublished data)
**29,30**	StAx-35, StAx-35R	Pull down assay. Competitive binding to Immobilized GST-tagged CBD of TCF4(1–52).
**31**	Carnosic acid	His-HD2 to glutathione S-transferase (GST)-ARD (immobilized on glutathione-coated microplates),
**32**	Pyrrolidinio	Alpha screen, ITC
**33**	hsBCL9CT-24	LEF/TCF reporter assay robust ALPHA competition assays were developed to determine the potency of inhibitors in disrupting the b-cat/BCL9 interaction.

Table S2. Summary of the activitiies of compounds **1–33.**

**Comp**	Label	Assay
**1**	FH535	Low micromolar LC_{50} values as Inhibitors of colon, lung and liver carcinomas expressing high or active Wnt/b-catenin, antagonist of PPAR◎ and PPAR.
**2**	GW9662	Specific PPAR◎ antagonistic, unable to antagonize PPAR◎ and to inhibit the Wnt/◎-catenin pathway.
**3**	FH535 analog	Superior to FH535 in the [^{3}H]-thymidine incorporation assay, weak activity in the TOPFlash assay.
**6**	FH535 analog	Weak activity in the [3H]-thymidine incorporation assay, comparable to FH535 in the TOPFlash assay.
**7**	MSAB	Decreased cell viability of Wnt-dependent HCT116, HT115 and H23 cells; inhibited tumor growth in xenograft mice model after 2 weeks treatment at 10–20 mg/kg.
**8–9**	MSAB analogs	Inhibition of Wnt reporter with an IC_{50} value of 7.0 μM, reducion the c-Myc levels, inhibition of HCT116 with IC_{50} of 20.2 μM.
**10**	LF3	IC_{50} of 1.65 and 1.82 ◎M in the AlphaScreen and ELISA assays.
**11**	LF3 analog	Showed inhibitory activity as potent as that of LF3 in the AlphaScreen and ELISA assays.
**12–14**	iCRTs	Selective disruption β-catenin/Tcf4 interactions; efficacy comparable with 5-FU in human CRC
**15**	ZINC02092166	Inhibition the TOPFlash luciferase activity in pcDNA3.1-β-catenin transfected HEK293 and SW480 cells (IC_{50} of 0.86 and 0.71 μM); SW480, HCT116, and HT29 cell growth inhibition with low micromolar IC_{50} values.
**16,17**	ZINC analog	Similar to that of ZINC02092166 In the AlphaScreen and FP assays.
**16,17**	ZINC analog	IC_{50} of 26 ◎M in the TOPFlash and FOPFlash luciferase reporter assay; i SW480 and HCY116 cancer cell growth inhibition with IC_{50} of 2.0 and 31 M.
**18**	PNU-74654	Kd of 450 nM in direct binding ITC experiments; specific inhibition for Tcf-4 transactivation in the cellular luciferase reporter system.
**19**	UU-T01	Ki of 3.14 ◎M as disruptor of ◎-catenin/Tcf interactions. ITC, Kd of 0.531 ◎M to WT -catenin, AlphaScreen Ki of 7.60 ◎M
**20**	UU-T02	Ki value of 1.32 μM against the WT ◎-catenin; complete disruption of the β-catenin/Tcf PPI.
**21**	UU-T03	IC_{50} of 28.7 mM in SW480 cells and IC_{50} of 37.6 ◎M in Wnt-activated HEK293 cells in the Wnt-responsive luciferase reporter assay.
**22**	HI-B1	β-catenin/Tcf4 interction inhibition in vitro in DLD-1 and Caco-2 cell lines; Reduction of growth of a PDX colon cancer with a high expression level of β-catenin.
**23–25**	PKF	Inhibition of Tcf4/◎-catenin association IC_{50} values of 2, 4, 0.8 and 0.64 mM, respectively.
**26**	PKF	In MM blocked expression of Wnt target genes and induced cytotoxicity, In xenograft models of hMM, inhibited tumor growth and prolonged survival.
**27**	Henryn	Reduced the expression of Cyclin D1 and c-Myc, and induced G1/S phase arrest in HCT116 cells by impairing the association of β-catenin/Tcf4.
**28**	BC21	Inhibitor of the β-catenin/Tcf4 driven luciferase activity. Decreased the HCT116 cell viability; at 5 ◎M inhibited >80% colony forming activities

**29,30**	StAx-35, StAx-35R	Both fStAx-35 and -35R suppressed luciferase activity. Proliferation of DLD1 and SW480 cells was blocked by aStAx-35R.
**31**	Carnosic acid	inhibited the binding of β-catenin to BCL9 in vitro, and β-catenin-dependent transcription in CRC cell
**32**	Pyrrolidinio	Selectively disruption β-catenin/BCL9 in cells overexpressing the Wnt signaling with IC_{50} values in the low micromolar range
**33**	hsBCL9CT-24	Kd value of 4.21 nM In the HTRF binding assay. Kd value of 4.73 nM in disrupting the ◎-catenin/BCL9 interaction.
